# A Right-Angled Thorn in the Bowel: A Curious Case of Small Bowel Perforation

**DOI:** 10.7759/cureus.44068

**Published:** 2023-08-24

**Authors:** Debajyoti Mohanty, Dharmendra Dugar, Asish Waliya

**Affiliations:** 1 General Surgery, All India Institute of Medical Sciences, Raipur, Raipur, IND

**Keywords:** emergency, laparotomy, small bowel, perforation, foreign body

## Abstract

We present a 45-year-old man with small bowel perforation resulting from the inadvertent ingestion of a right-angled thorn of the Gum Arabic plant (Vachellia nilotica). The diagnosis was made, and an emergency laparotomy was performed for suspected enteric peritonitis. The thorn was found projecting from the terminal ileum with a minimal intra-peritoneal fluid collection. The thorn was removed, and the perforation site was repaired primarily with absorbable sutures. The lack of a reliable history of foreign body ingestion makes it impossible to arrive at an accurate preoperative diagnosis in patients presenting with perforation peritonitis. Radiological investigations have a low sensitivity for detecting radiolucent vegetative foreign bodies as the cause of bowel perforations. Primary repair should be preferred over resection procedures in the management of foreign body-induced small bowel perforations.

## Introduction

Small bowel perforations account for a significant proportion of patients presenting with acute abdomen [1]. Most of these perforations are secondary to ulcerative bowel pathologies such as typhoid, tuberculosis, inflammatory bowel disease, and malignant neoplasm, while perforations secondary to blunt and penetrating abdominal trauma are relatively less frequent [2]. Traumatic gastrointestinal tract (GIT) perforation by an ingested foreign body (IFB) is uncommon and reported in less than 1% of patients [3]. Sharp bony fragments are the culprit in more than half of these patients, while bowel perforation resulting from vegetative foreign body ingestion is infrequent [4]. We herein report a patient having small bowel perforation following accidental ingestion of a right-angled thorn of the Gum Arabic plant.

## Case presentation

**Figure 1:**
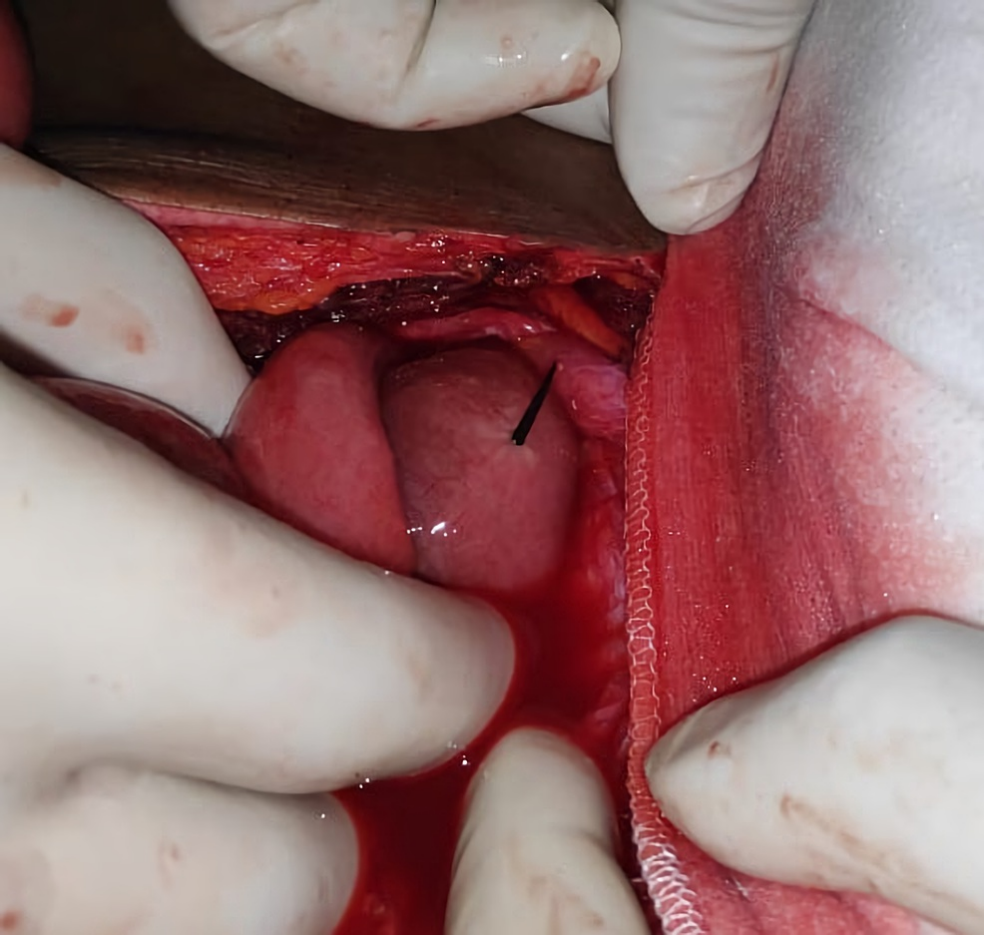
Protruding foreign body from the ante-mesenteric border of the ileum

A 45-year-old Caucasian man presented to the emergency department with severe generalized abdominal pain, low-grade fever, and a reduced appetite of five days duration. The patient had received conservative treatment for his complaints at a local hospital before being referred to our centre due to worsening symptoms. The patient was febrile to touch with a temperature of 100.2 degrees Fahrenheit, pulse rate of 100 beats/ min, respiration rate of 20/ min, and blood pressure of 118/84 mm Hg. Signs of peritonism, such as central abdominal distension, marked periumbilical tenderness, and rebound tenderness, are evident on abdominal examination. Haemoglobin was 11.4 mg/dl (13.0-17.0 mg/dl); total leukocyte count was 10,500/ cu mm (4,000-11,000 cu mm) with 75% neutrophils (range 40%-75%) and 11% eosinophils (range 1%-6%). The liver and kidney functions were normal. Serology for HIV antibodies was non-reactive. The chest X-ray PA view revealed free gas under the right dome of the diaphragm. An abdominal contrast-enhanced computed tomography (CECT) scan of the patient documented the presence of intramural air in the small bowel loops, suggestive of mesenteric ischemia, and subdiaphragmatic air in the right side over the liver, suggestive of perforation. Based on the clinical signs and imaging findings of free gas under the diaphragm, a working diagnosis of perforation peritonitis was made. The patient was kept nil per oral with nasogastric tube aspiration, and intravenous fluid resuscitation with crystalloid solutions was initiated. Prophylactic antibiotics were administered in the form of intravenous ceftriaxone 1g 12 hourly and intravenous paracetamol 1g 8 hourly for analgesia. The patient was subjected to an emergency midline laparotomy. A dark colour thorn was found projecting from the ante-mesenteric border of the ileum about 30 cm proximal to the ileocaecal junction during the survey of the peritoneal cavity (Figure 1).

**Figure 2:**
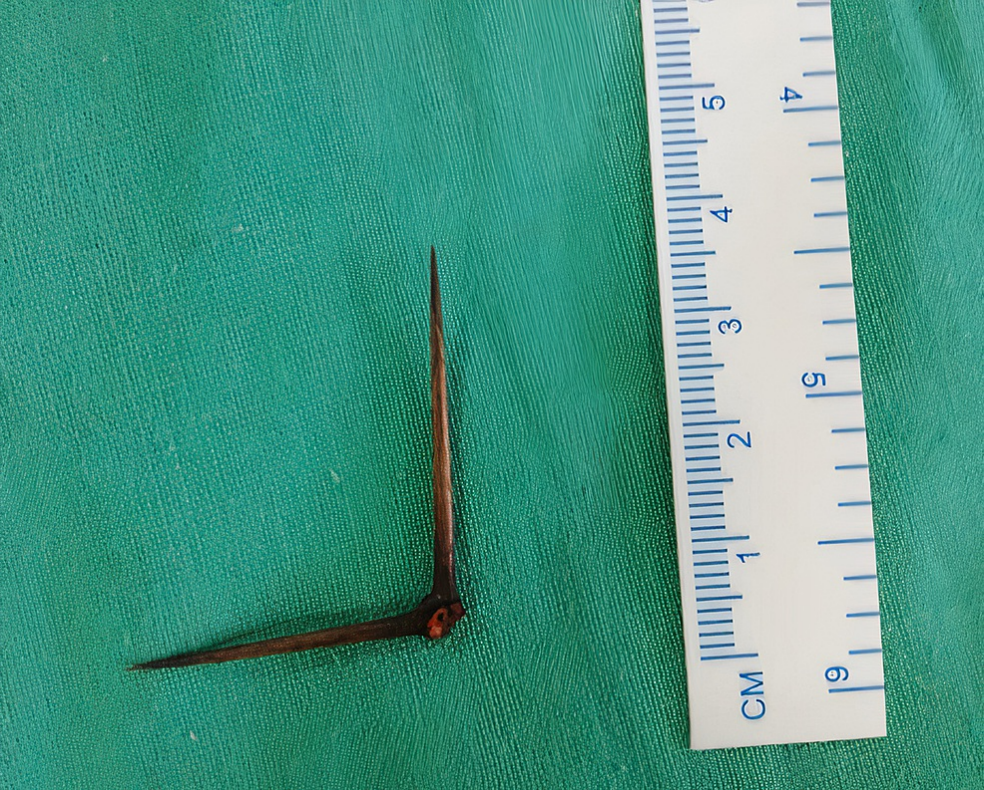
Retrieved right-angled thorn of Gum Arabic plant

The adjacent bowel wall was edematous with minimum intra-peritoneal contamination. The thorn was removed, and the bowel penetration site was repaired with a 4-0 PDS suture. The retrieved right-angled thorn was 5 cm in size with sharp-pointed ends (Figure 2).

Primary skin closure of the laparotomy incision was performed after thorough peritoneal lavage and placement of an abdominal drain. When the recovering patient became aware of the thorn as the cause of bowel perforation, he recalled experiencing sudden pain in the throat with swallowing difficulty while having a spicy Indian dish containing battered and deep-fried chopped vegetables during dinner 10 days back. The patient also identified the retrieved object as a thorn of the Babul or Gum Arabic plant (Vachellia nilotica) abundant in his locality. The postoperative recovery was uneventful, without evidence of surgical site infections. The patient was discharged on the eighth postoperative day following suture removal. The patient was well on follow-up one year after discharge.

## Discussion

Small bowel perforation is relatively common in patients presenting with acute abdomen to surgical emergency in the Indian subcontinent. Most of these perforations are secondary to ulcerative pathologies such as typhoid, tuberculosis, and inflammatory and malignant bowel disease [2]. Traumatic perforations by ingested and impaled foreign bodies are uncommon and reported in less than 1% of patients [3]. Erosion and bleeding, obstruction, perforation, intraabdominal abscess, and internal fistulization are the usual intestinal complications following ingestion of foreign bodies [5]. Extraintestinal complications can either be secondary to direct IFB penetration of viscera adjacent to the gastrointestinal tract (GIT), such as the liver, pancreas, ureter, kidney, blood vessels, heart, and vertebra, or secondary complications of IFB-induced perforation peritonitis, such as portal pyemia, septicemia, and multiple organ dysfunction syndrome [3,4,6]. 

The known risk factors for foreign body ingestion are extremes of age, poor eyesight, ill-fitting dentures, psychiatric disorders, decreased consciousness level as in alcoholics, drug abusers, debilitating illness, and overzealous binge eating episodes. Foreign bodies having a large size, rigid structure, sharp margin, and corrosive potential are more harmful than small, malleable, blunt-ended inert objects. Physiological gut constrictions and angulations, junction of mobile and fixed parts of the bowel, and transition zones resulting in a change in consistency of luminal contents are the potential sites for entrapment of the ingested foreign bodies. The duodenum, gastric antrum, ileocaecal and rectosigmoid regions are the most vulnerable to impaction and perforation by ingested foreign bodies [7]. In addition, pre-existing bowel ulceration, stricture, stenosis, diverticulum, and adhesion also enhance the risk of IFB-induced complications. 

The patients of accidental foreign body ingestion have a varied clinical presentation depending on the site of foreign body impaction. The spectrum of the presentation includes abdominal pain, anal pain, parietal abscess, constipation, anal fistula, and abdominal and systemic sepsis [8]. Of all the symptoms related to IFBs, abdominal pain is encountered in 95% of the patients, fever in 81% and peritonitis in 39% [9]. While most IFBs are extruded within a week of ingestion, patients with IFB-induced bowel perforation usually have a delayed presentation with ingestion to a perforation interval of 10.4 days [10]. The plausible explanation can be that an intense inflammatory reaction invariably accompanies the gradual bowel wall penetration by the impacted sharp object. The consequent inflammatory bowel oedema produces a tight grip on the offending foreign body, preventing extra-luminal spillage of intestinal contents. Adhesions formed by the fibrin deposits, omentum, and adjacent bowel loops also limit the spread of contamination. 

Preoperative diagnosis of IFB-induced bowel perforation is seldom made in patients with peritonitis due to their failure to recollect any such episode of ingestion and lack of reliable radiological signs suggestive of intraluminal foreign body. Imaging does play an essential role in identifying the location, size, and shape of the radio-opaque foreign body in patients with a definite history of ingestion. Serial plain abdominal roentgenograms are often relied upon for documenting the foreign body progression through the gut in these patients. An ultrasound scan and CECT of the abdomen are essential in symptomatic patients of foreign body ingestion. The sensitivity of the CECT of the abdomen in detecting sharp nonmetallic foreign bodies such as toothpicks is reported to be only 42.6% [11]. Both ultrasound and magnetic resonance imaging (MRI) are helpful in imaging ingested wooden foreign bodies with a sensitivity ranging from 52% to 82% [12]. These imaging modalities contribute to the diagnosis by precisely identifying the high density of foreign body shadows, associated pneumo-peritoneum, intra-abdominal collection, bowel wall thickening, increased mesenteric fat density, and inflammatory phlegmon [6,13]. 

Sharp pointed ends, corrosive potential, impaction evident by lack of progression over 48 to 72 hours, and GIT complications are the usual indications for intervention. The location of the IFB and the mode of clinical presentation guide the selection of a suitable approach for retrieval. Endoscopic removal is preferred for foreign bodies lodged in the upper GIT; however, it has limited scope in retrieving small bowel foreign bodies due to restricted access and technical complexity. The subset of patients with apparent signs of peritonitis will require surgical management. Delaying surgical intervention for an endoscopic retrieval attempt in overt peritonitis patients is not recommended [14]. Depending on the available expertise, surgical retrieval of small bowel foreign bodies can be accomplished by both open and minimal access techniques. Primary repair of bowel perforation or limited resection should be favoured over extensive resections due to the benign nature of the pathology. The decision for ostomy formation is prudent in patients having poor nutritional and performance status and gross peritoneal contamination with edematous bowel. The small size of the perforation, scant peritoneal contamination, and absence of pre-existing bowel disease in our normotensive patient favoured simple suture repair of the defect over bowel resection for managing the small bowel perforation [2].

## Conclusions

In conclusion, the configuration of the IFB is critical for developing GIT complications, with large and pointed foreign bodies more likely to impact and give rise to GIT complications. In most cases, unreliable history and nonspecific radiological signs are responsible for the missed preoperative diagnosis. The patients of IFB should be managed according to their pathogenesis. Those patients with no evidence of bowel obstruction or perforation should be managed conservatively with an expectation for spontaneous expulsion of the foreign body, while conservative surgery is adequate in managing intestinal perforations associated with IFB.
